# *Human papillomavirus* type 16 E6 and E7 oncoproteins interact with the nuclear p53-binding protein 1 in an in vitro reconstructed 3D epithelium: new insights for the virus-induced DNA damage response

**DOI:** 10.1186/s12985-018-1086-4

**Published:** 2018-11-16

**Authors:** Diletta Francesca Squarzanti, Rita Sorrentino, Manuela Miriam Landini, Andrea Chiesa, Sabrina Pinato, Francesca Rocchio, Martina Mattii, Lorenza Penengo, Barbara Azzimonti

**Affiliations:** 1 Laboratory of applied Microbiology, Department of Health Sciences, University of Piemonte Orientale (UPO), Via Solaroli 17, 28100 Novara, Italy; 2 Laboratory of Biomedical Materials, Department of Health Sciences, University of Piemonte Orientale (UPO), Novara, Italy; 3 Laboratory of Molecular Biology, Department of Pharmaceutical Sciences, University of Piemonte Orientale (UPO), Novara, Italy; 4 Institute of Molecular Cancer Research, University of Zurich, Zurich, Switzerland; 5 Consorzio Interuniversitario Nazionale per la Scienza e Tecnologia dei Materiali (INSTM, Firenze, Italy- Local Unit of Piemonte Orientale, UPO, Novara, Italy

**Keywords:** High-risk *Human Papillomavirus*, Cancer, DNA damage response, E6-associated protein, Double-strand break, In vitro 3D epithelial model, Proximity ligation assay, World Health Organization, Genomic instability

## Abstract

**Background:**

Despite vaccination and screening measures, anogenital cancer, mainly promoted by HPV16 oncoproteins, still represents the fourth tumor and the second cause of death among women.

Cell replication fidelity is the result of the host DNA damage response (DDR). Unlike many DNA viruses that promote their life cycle through the DDR inactivation, HR-HPVs encourage cells proliferation despite the DDR turned on. Why and how it occurs has been only partially elucidated.

During HPV16 infection, E6 links and degrades p53 via the binding to the E6AP LXXLL sequence; unfortunately, E6 direct role in the DDR response has not clearly identified yet.

Similarly, E7 increases DDR by competing with E2F1-pRb interaction, thus leading to the inactivation of pRb, and promotion, E2F1 mediated, of DDR genes translation, by binding to the pRb-like proteins CBP/p300 and p107, that also harbour LXXLL sequence, and via the interaction and activation of several DDR proteins.

**Methods:**

To gain information regarding E6 and E7 contribution in DDR activation, we produced an in vitro 3D HPV16-E6E7 infected epithelium, already consolidated study model for HPVs, and validated it by assessing H&E staining and BrdU, HPV16 DNA, E6E7 proteins and γH2A.X/53BP1 double-strand break (DSBs) sensors expression; then we made an immuno-colocalization of E6 and E7 with cyclin E2 and B1.

Since 53BP1, like E6 and E7, also binds p53 and pRb, we supposed their possible direct binding. To explore this hypothesis, we performed a double immunofluorescence of E6 and E7 with 53BP1, a sequence analysis of 53BP1 within its BRCT2 domain and then an in situ PLA within CaSki, E6E7HPV16 NHEKs and the 3D model.

**Results:**

The in vitro epithelium resembled the histology and the events typical of in vivo infected tissues. E6E7HPV16 were both expressed in basal and differentiated strata and induced H2A.X phosphorylation and 53BP1 increment into nuclear foci.

After highlighting E6 and E7 co-expression with 53BP1 and a LKVLL sequence within the 53BP1 BRCT2 domain, we demonstrated the bindings via the PLA technique.

**Conclusions:**

Our results reinforce E6 and E7 role in cellular function control providing potentially new insights into the activity of this tumor virus.

## Background

*Human papillomavirus* (HPV) family groups a heterogeneous number of viruses able to infect squamous stratified epithelia and cause benign papillomas, warts and anogenital lesions, depending on the viral genotype, time of persistence and possible integration into the eukaryotic host genome. The virus also correlates with oropharyngeal malignancies, strongly rising because of sexual behaviors changes [1].

HPV vaccine, the first one developed in 1991, till now represents, with its 2-, 4- and 9-valent formulations, the best way to prevent and control both infection and genital cancer progression that are mainly mediated by high risk HPV16 and 18 oncoproteins. Ten years of phase III clinical trials revealed their optimal effectiveness with success rates ranging from 90 to 100%, although to be confirmed by a 20 years overall period of clinical evidences [2, 3].

Despite this and the strict screening measures, anogenital cancer, mainly promoted by the over-expression of the high risk α-HPV16 E6 and E7 oncoproteins which destabilize the host genome over time after primary infection [4–6], still represents the fourth most common tumor and the second largest cause of death among women in the world, with about more than 5 hundred thousand new cases/year, as evidenced by the World Health Organization (WHO).

Cell replication fidelity is the result of the host DDR. Unlike many DNA viruses that promote their life cycle through the DDR inactivation, HR-HPVs encourage cells proliferation despite the DDR turned on. Why and how it occurs has only partially elucidated.

Although a lot of research has been published in the area [7–9], further insights are needed to deepen the replication and host genome destabilizing properties of the virus, from the early phases of the tumorigenesis process, in order to discover new target therapies for unvaccinated HPV infected patients and for those in whom vaccination is not the right approach.

Well established reports demonstrate that HR-HPVs can efficiently amplify their genome thanks to the independent, but cooperative ability of E6 and E7 oncoproteins to manipulate the DDR and repair machinery of the targeted host’s epithelial cells [10–14], activated just after the oncogene-induced replicative stress, leading to genomic instability (GIN) and cancer progression [15–18].

It is well known that HR-HPV16 E6 oncoprotein is highly expressed during the early phases of infection and responsible for p53 degradation, via the proteasome machinery, through the binding with E6AP, a HECT domain ubiquitin ligase [19]. Both E6 and E6AP alone are unable to bind p53, suggesting that their interaction is crucial for p53 degradation [20–22]; indeed, structure analysis reveals that such binding induces conformational changes in E6AP that allow the link with p53 [23].

The E6AP binding relies on a short leucine rich aminoacidic sequence, called LXXLL binding motif; when deleted and/or mutated, the formation of the E6-E6AP complex is prevented, thus failing to target p53 for degradation [21, 23–25].

Similarly, E7 increases DDR through several mechanisms: by competing with E2F1-pRb interaction, thus leading to the inactivation of pRb, and promotion, E2F1 mediated, of DDR genes translation, by binding to the pRb-like proteins CBP/p300 and p107, that also harbour LXXLL sequence, and via the interaction and activation of several DDR proteins.

Because of E6 and E7 interaction with several LXXLL binding motif containing proteins [26–30], we hypothesized their binding also to one of the LXXLL motifs contained into DNA damage sensors.

On these bases, we show experimental evidences to support our theory by using an in vitro reconstructed 3D infected epithelium [31–34], made of HPV16E6E7 transduced keratinocytes and already consolidated for HPVs study, as a surrogate of an in vivo lesion. H/E and immunofluorescence stainings for the main viral markers (HPV16-DNA, E6 and E7), DSBs sensors γH2A.X and 53BP1 and cyclins (E2 and B1) were firstly made in order to reproduce, inside the 3D model, what in vivo happens.

Afterwards, despite the existence of many DNA damage sensor targets such as 53BP1, well-recognized DDR mediator/adaptor, MDC1 (mediator of DNA Damage Checkpoint 1), BRCA1 (Breast Cancer 1, early onset), TOPBP1 (Topoisomerase II-binding protein 1) and Claspin, we supposed that E6 and E7 could directly interfere just on 53BP1 activity since they all respectively link to p53 and pRb for the control of anti-tumorigenic cell-fate decisions and for their rapid proteasome-mediated degradation and inactivation respectively.

After highlighting E6 and E7 co-expression with 53BP1 by IF analysis, [35–41] and found the presence of a LXXLL binding motif within the 53BP1 BRCT2 domain (LKVLL), we finally evidenced the complex and direct interaction between E6 and E7 HPV16 oncoproteins with 53BP1 via the highly specific and sensible in situ PLA system [42–46], well known and validated assay able to identify and characterize interactions in native proteins in their correct tissue/cell context under near natural/physiological conditions.

## Methods

### Normal and HPV16 positive neoplastic epithelia

Normal epithelia, obtained by the tumor-free margins of informed consent patients who underwent surgical procedures, and HPV16 positive anogenital intraepithelial neoplasia (AIN, grade III), selected by the records of the Pathology Unit of the Azienda Ospedaliero-Universitaria “Maggiore della Carità” of Novara, Italy, were used as the in vivo counterpart of the 3D models.

### 2D cell cultures

Authenticated pooled neonatal normal human epidermal keratinocytes (NHEKs) were obtained from Lonza (distributed by Euroclone, Milan, Italy) and used to produce, by lentiviral infection, LacZ and E6E7 HPV16 keratinocytes (control normal and E6E7HPV16 NHEKs) as previously described by Azzimonti et al. [47]. Epithelial cells were grown in monolayer in EpiLife® Medium (Invitrogen, Milan, Italy).

Certified human dermal fibroblasts (HDFs) were bought from ATCC (distributed by LGC Standards, Sesto San Giovanni, Milan, Italy) and maintained in DMEM supplemented with 10% heat inactivated fetal bovine serum (FBS) both from Sigma Aldrich (Milan, Italy) at 37 °C in a humidified 5% CO_2_ atmosphere.

CaSki cell line, reported to contain an integrated HPV16 genome (about 600 copies per cell) [48], was also obtained by ATCC and maintained as above described.

### 3D in vitro epithelial cultures establishment

**Fig. 1:**
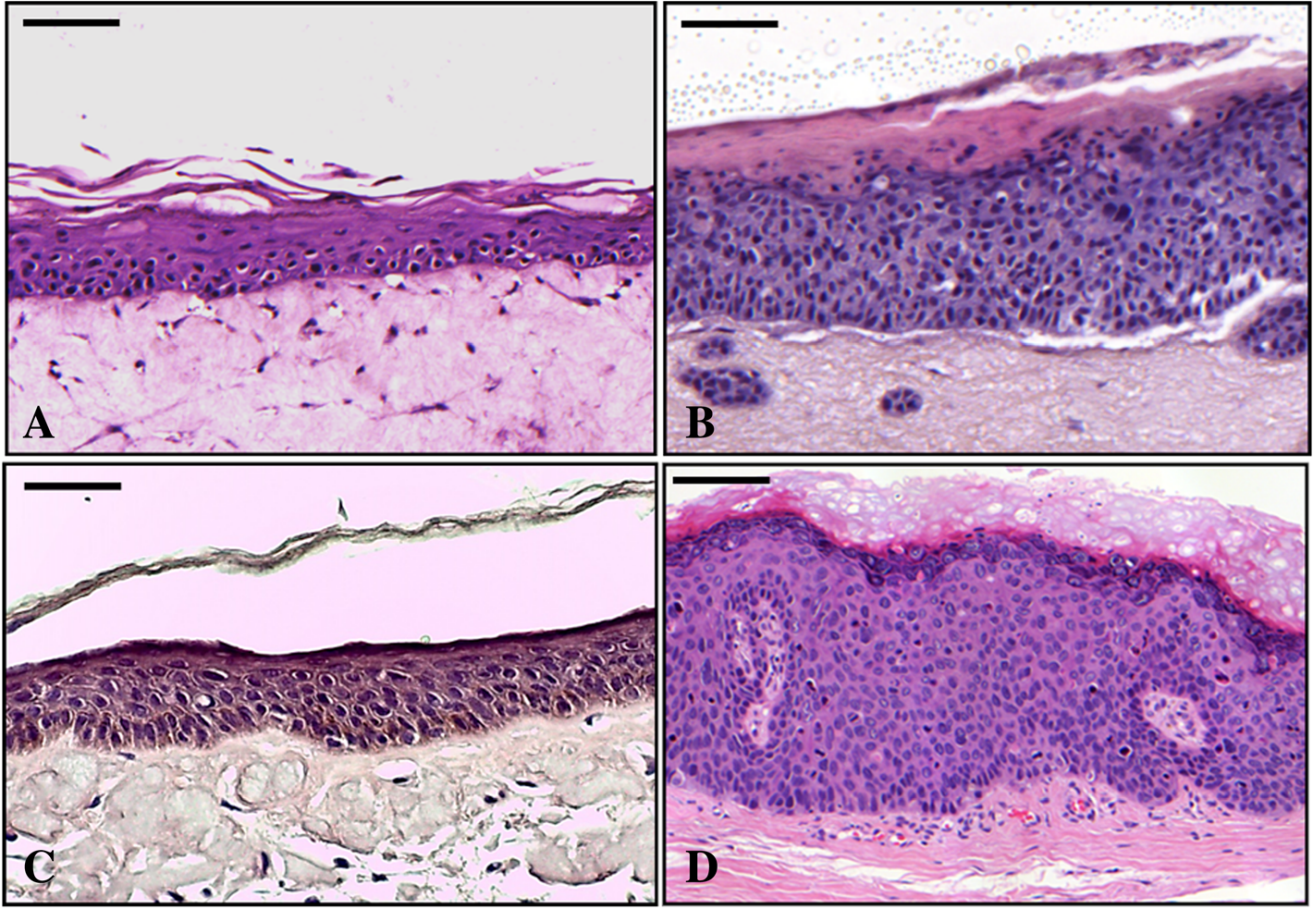
H&E staining. **A**) Control in vitro reconstructed 3D epithelial culture made up with normal keratinocytes and **B**) E6E7 HPV16 transduced keratinocytes. **C**) In vivo normal epithelia obtained from tumor-free mucosa margins. **D**) HPV16 positive anal intraepithelial neoplasia (AIN), grade III. Bar scales in 20x fields = 100 μm

3D epithelial cultures were prepared as previously described [47, 49, 50], with some modifications. Briefly, normal and E6E7HPV16 NHEKs (1x10^6^cells), produced as shown in the 2D culture paragraph, were seeded onto type I rat tail collagen plugs embedded with HDFs and kept submerged for four days. Cultures were then raised at the air-liquid interface for 14 days, with culture medium change every other day. To mark cells in S phase, eighteen hours before harvesting they were exposed to BrdU (5′-bromo-2′-deoxyuridine, 100μg/ml, Sigma Aldrich). In vivo and in vitro normal and HPV16 positive specimens were fixed in 10% buffered formalin, embedded in paraffin, cut into 4-μm-thick sections, placed onto Superfrost ultra plus glass slides (Menzel Glaser, distributed by BioOptica, Milan, Italy) and counterstained by hematoxylin and eosin (H&E; Sigma Aldrich; Fig. 1A-D).

### 3D in vitro epithelial cultures analysis

#### HPV16 DNA fluorescent in situ hybridization (FISH) and BrdU stainings

HPV16 DNA detection was performed onto the normal and HPV16 positive 3D tissues as described by Peh et al. [51]. Complete genomic plasmid HPV16 DNA probe (cloned in pBR322 vector, kindly obtained by the Karolinska Institute, International HPV Reference Center, Forskningsgatan, Stockholm, Sveden) was biotin labeled (Biotin NT Labelling Kit, Jena Bioscience, Borsea, Rovigo, Italy) according to the manufacturer’s instructions. Deparaffinized and rehydrated sections, after target denaturation in 50 μg/ml proteinase K for 20 min at 37 °C and unmasking in EDTA 1 mM pH 8 for 50 min at 95 °C, were heated for 5 min at 95 °C with the BIO-labeled HPV16 DNA probe and incubated overnight (ON) at 37 °C.

**Fig. 2:**
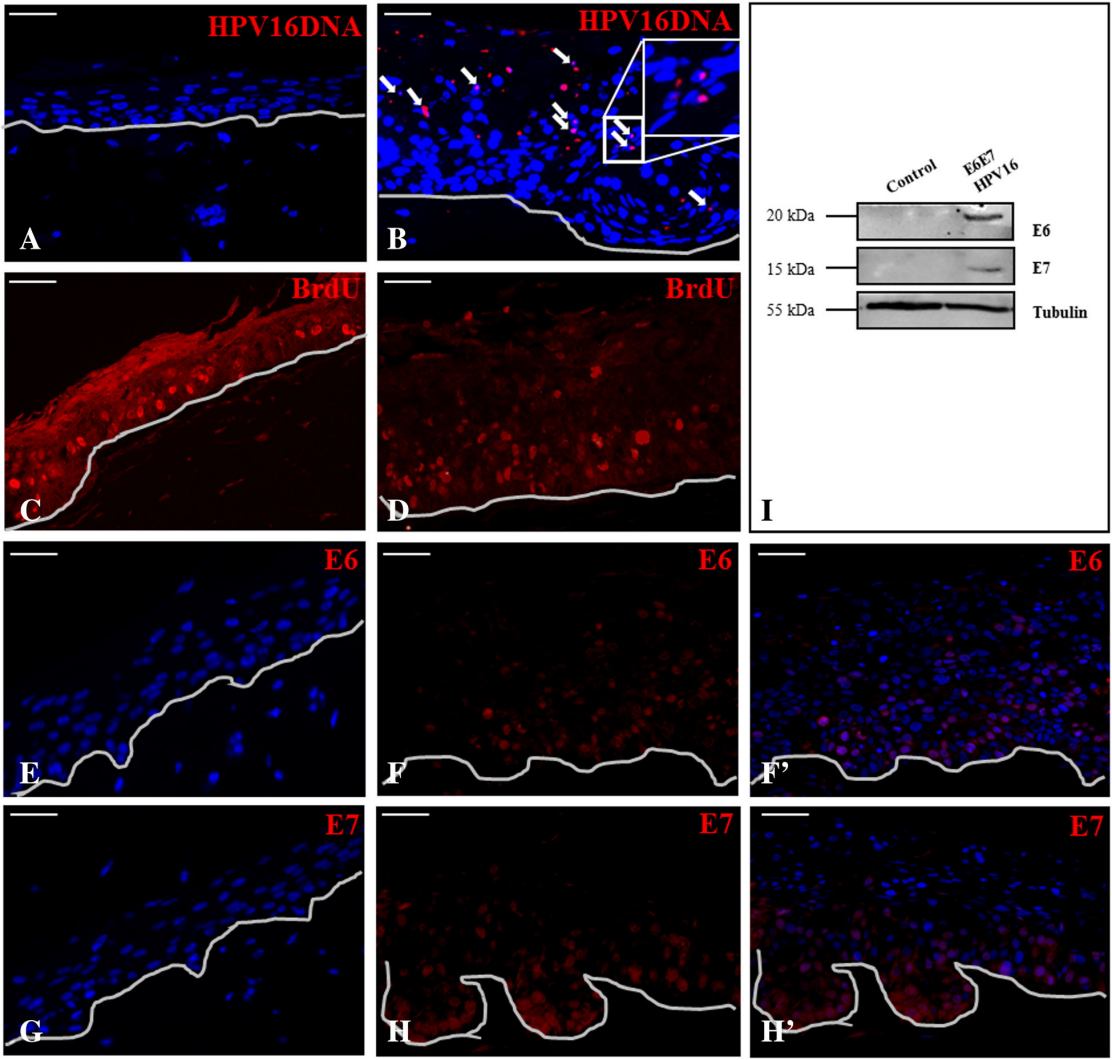
In vitro 3D epithelial cultures. HPV16 DNA (FISH) staining and immunofluorescent detection of BrdU, E6 and E7 of control normal and E6E7 HPV16 infected reconstructed in vitro 3D epithelial cultures. Viral genome amplification positivity is evidenced in red in the mid and upper strata of the infected epithelium only (**A**, **B**). To show cell nuclei, sections are counterstained with DAPI (blue). S phase cells positivity to the BrdU marker is limited to the basal and basal/parabasal strata of normal (**C**) and infected epithelia (**D**) respectively. Direct analysis of E6 and E7 proteins in normal uninfected (**E** and **G** for E6 and E7 respectively) and E6E7 HPV16 positive culture sections (**F**-F′ for E6 and **H**-H′ for E7). Positive cells are shown in red, while cells nuclei are counterstained in blue with DAPI. Bar scales in 20x fields = 100 μm. Immunoblotting. Western blot analysis of E6, E7 and tubulin protein extracts derived by peeling off the epithelial layers of 3D reconstructed epithelial cultures (control normal and E6E7HPV16) from the collagen-based dermis. After run on polyacrylamide gels, proteins were blotted onto nitrocellulose or PVDF membranes (**I**)

After stringent washes, sections were blocked for 1 h at room temperature (RT) with 1% bovine serum albumin (BSA) plus 0.2% gelatin from cold water fish skin in PBS 1x (all from Sigma). BIO-labeled DNA was revealed by using an ABC kit (Vector Laboratories, distributed by DBA Italia Srl, Segrate, Italy) followed by a Tyramide Signal Amplification kit (TSA, Perkin Elmer, distributed by Thermo Scientific, Milan, Italy), according to the manufacturer’s instructions. HPV DNA positive cells were evidenced in red, while cell nuclei were counterstained in blue with DAPI (Fig. 2A-B).

For BrdU epitope retrieval, a 2 N HCl treatment, followed by a 1:200 diluted primary mouse monoclonal anti-BrdU antibody (BU-33, Sigma Aldrich) incubation (both for 1 h at 37 °C) was performed. BrdU was detected with an Alexa Fluor 488 dye conjugated specific secondary antibody (Fig. 2C-D).

#### Viral proteins expression

To assess HPV16 oncoproteins expression inside the 3D in vitro epithelium, a direct immunofluorescent and a western blot analysis were performed. Uninfected normal and E6E7 HPV16 positive 3D cultures were ON incubated at 4 °C with the following monoclonal mouse primary antibodies: anti HPV16/18 E6 (clone C1P5, working dilution 1:100, Santa Cruz Biotechnology, distributed by DBA Italia, Segrate, Milan, Italy) and anti HPV16 E7 (clone ED17, working dilution 1:50, Santa Cruz Biotechnology). For antigens retrieval, all sections were incubated in 1 mM EDTA pH 8 for 50 min at 95 °C. Both E6 and E7 incubations were followed by the addition of the MACH3 mouse HRP-polymer (BIOCARE Medical, distributed by Space Import Export, Milan, Italy), according to the manufacturer’s instructions, and TSA signal development (Fig. 2E-F-F’-G-H-H′).

Protein extracts were obtained by peeling off the epithelial layers of the reconstructed epithelium from the collagen based dermis, then cut into small pieces with a surgeon knife, subjected to 3 sonication cycles and finally lysed in RIPA buffer (0.1 M Tris-HCl pH 7.4, 0.3 M NaCl, 2% Triton X100, 2% Sodium dehoxycholate and 0.2% SDS) plus inhibitors (1 mM PMSF, 0.5 mM Sodium pirofosphate, 50 mM Sodium fluoride, 0.2 mM Sodium orthovanadate, protease inhibitor cocktail and 50 U/μl Benzonase).

Sixty μg of protein extracts were run onto 10% and/or 15% SDS-polyacrilamide gels and then transferred onto nitrocellulose (GE Healthcare, Milan, Italy) and/or PVDF membranes (EMD Millipore Corporation, Milan, Italy). Membranes were probed with the following primary antibodies: anti-E6 (working dilution 1:500) and anti-E7 (working dilution 1:300). A mouse anti**-**tubulin antibody (working dilution 1:2000, clone DM1A, abcam) was used as internal protein loading normalization control (Fig. 2I). A peroxidase goat anti-mouse antibody (working dilution 1:2000, abcam) incubation was followed by antigens detection using an enhanced chemiluminescence kit (SuperSignal West Pico Chemiluminescent Substrate, Thermo Fisher Scientific, Rockford, IL, USA). Images were acquired by the Quantity One software (version 4.6.9; Bio-Rad Laboratories, Segrate, Milan, Italy).

### Characterization of the DDR in the 3D in vitro HPV16 epithelial models

#### DSBs sensors protein-protein detection

Uninfected normal and E6E7 HPV16 positive 3D cultures were processed by both IF and biochemical analysis.

In vitro cultures slides were ON incubated at 4 °C with the following primary antibodies: mouse anti-γH2A.X (clone JBW301, working dilution 1:400, EMD Millipore Corp., USA, distributed by Merck, Darmstadt, Germany) and rabbit polyclonal anti-53BP1 (ab36823, working dilution 1:1000, abcam).

**Fig. 3:**
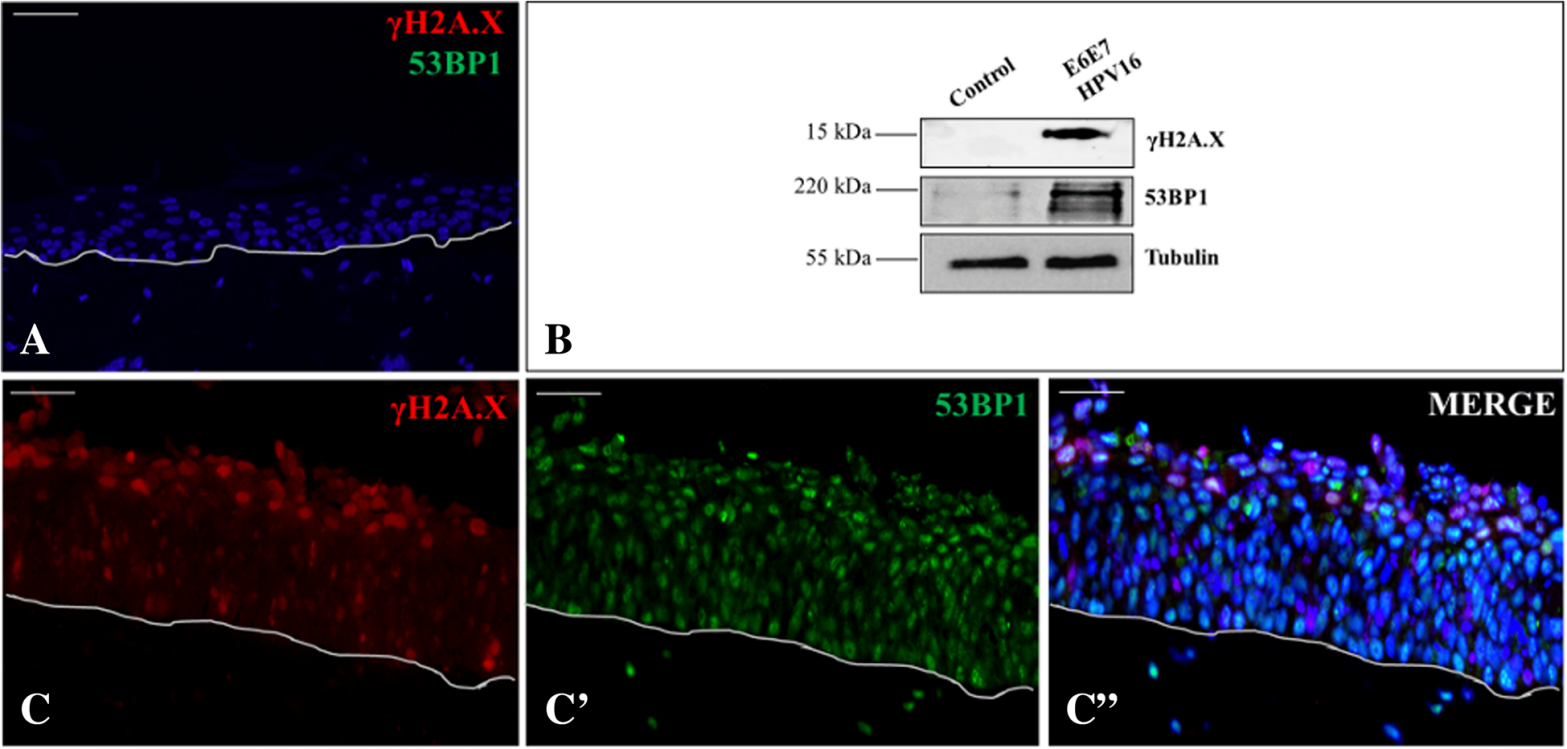
DDR characterization of the 3D in vitro reconstructed E6E7 HPV16 infected epithelia. Double immunofluorescent and western blot (**B**) analysis of the DNA break sensors. γH2A.X (in red) and 53BP1 (in green) in control normal (**A**) and E6E7HPV16 infected epithelia (**C**-C′-C″). γH2A.X and 53BP1 are expressed in HPV infected 3D samples

For antigens retrieval, all sections were incubated in 1 mM EDTA pH 8 for 50 min at 95 °C. γH2A.X and 53BP1 incubations were respectively followed by the addition of Alexa Fluor 568 and 488 dyes conjugated specific secondary antibodies, according to the manufacturer’s instructions, and TSA signal development (Fig. 3A-C, C’, C″).

Protein extracts were then obtained, run, transferred and detected as above described. Membranes were probed with the following primary antibodies: anti-γH2A.X (working dilution 1:1000), anti-53BP1 (working dilution 1:1000) and anti-tubulin as internal control (Fig. 3B). A peroxidase-coupled goat anti-rabbit (working dilution 1:2000, abcam) or anti-mouse antibody (working dilution 1:2000, abcam) incubation was made. Antigens were then revealed as above described.

#### E6-E7/cyclin E2-cyclin B1 and E6-E7/53BP1 protein-protein detection

**Fig. 4:**
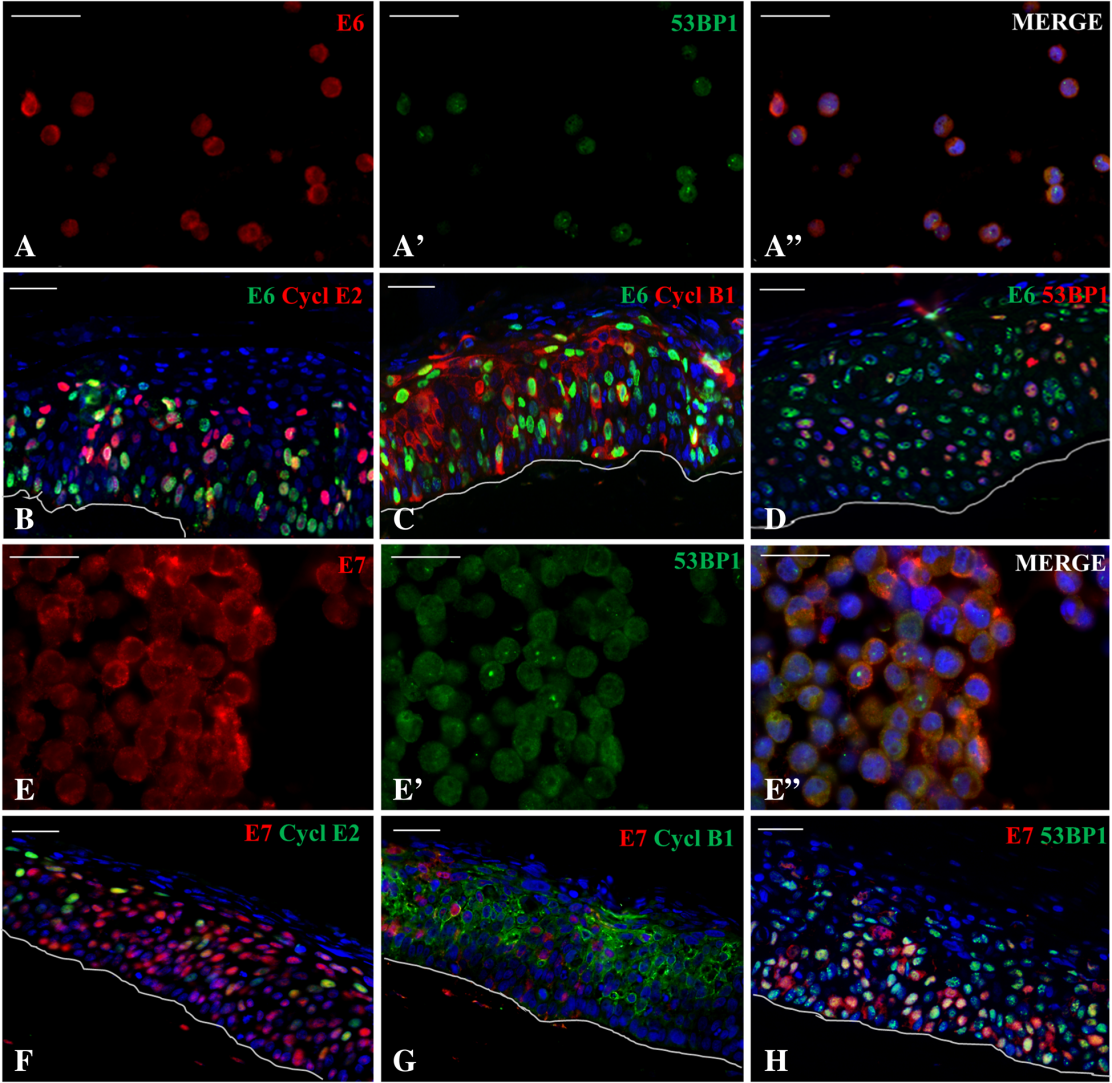
Double immunofluorescent analysis of E6 and E7 with cyclin E2 and B1. E6 and E7 HPV16 pushes a subset of differentiating cells into G1/S (cyclin E2 positive cells, **B** and **F**) and S/G2 checkpoint phases (cyclin B1 positive cells, **C** and **G**). Double immunofluorescent analysis of E6 and E7 with 53BP1. CaSki cells have a high number of discrete small 53BP1 positive nuclear foci inside most of the same cells that were E6 (**A**-A’-A”) and E7 (**E**, E’, E”) positive; in HPV16 epithelial models E6/53BP1 (**D**) and E7/53BP1 (**H**) positivity is throughout the epithelium, both in undifferentiated and differentiated layers. Nuclei were always visualized in blue with DAPI counterstaining. Bar scales in 20x fields = 100 μm

A double co-staining of E6 and E7 with cyclin E2 and B1 respectively (Fig. 4B-C for E6 and F-G for E7) was performed onto 3D epithelia. Antigens were respectively detected, after a 1 mM EDTA pH 8 unmasking for 50 min at 95 °C, by applying the anti-HPV16/18 E6 or anti HPV16 E7 with anti-Cyclin-E2 (Clone EP454Y; working dilution 1:250, abcam, distributed by Thermo Scientific, Milan, Italy) and Cyclin**-**B1 (Clone EPR17060; working dilution 1:250, abcam) monoclonal antibodies for 4 h at RT followed by an incubation with the respective Alexa Fluor-conjugated secondary antibodies (1:200, Molecular Probes, Oregon, USA) for 1 h at RT.

A double staining for E6 and E7 with 53BP1 (working dilution 1:200) was then setup (Fig. 4A-A’-A”-D for E6/53BP1 and Fig. 4E-E’-E”-H for E7/53BP1) onto CaSki cells and 3D culture sections.

To perform the proteins co-detection onto CaSki cells, they were grown until 95% confluence, detached by trypsin and centrifuged for 5 min at 900 rpm. Pellets were washed twice in PBS 1X, centrifuged for 10 min at 1200 rpm and resuspended in 100 μl of pre-warmed (50 °C) 2% agar solution and let solidify for 20 min at RT.

All immunostained sections were counterstained in blue with DAPI and mounted for image acquisition with a DM6000B inverted fluorescence microscope equipped with DFC350FX digital camera (Leica Microsystems, Milan, Italy).

### Sequence analysis

Aminoacid sequence of 53BP1 protein was acquired from the GenBank database, National Center for Biotechnology Information, https://www.ncbi.nlm.nih.gov/protein/1239290988. Sequence analysis of 53BP1 protein was made by BLASTP 2.8.0+ software [22].

### Immunofluorescent in situ proximity ligation assay (PLA)

Since PLA technology allows the in situ detection, with sub-cellular resolution and molecular biology precision (0–40 nm), of protein-protein interactions within tissues [42, 43, 52–58], we revealed E6 and E7 antigens binding with 53BP1 by circularized, ligated and amplified complementary and fluorescent labeled oligonucleotides bounded antibodies in the 3D model. Two negative controls were performed (data not shown) by: *i*) omission of the primary antibodies and *ii*) single antibody incubation. CaSki cells, used as positive control [59], were fixed, like E6E7HPV16 NHEKs, and paraffin-embedded as previously described.

Briefly, after EDTA unmasking, HPV16 positive 3D culture sections were incubated with Duolink blocking buffer (Duolink® In Situ Starter Kit Mouse/Rabbit, Sigma Aldrich) for 30 min at 37 °C, then ON at 4 °C with the specific primary antibodies (53BP1/E6 and 53BP1/E7) combinations. Sections were secondarily incubated with anti-rabbit PLUS and anti-mouse MINUS PLA probes (working ratio 1:5) for 1 h at 37 °C. Ligation was performed for 30 min at 37 °C and signals were amplified with Duolink In Situ Detection Reagent Red for 100 min at 37 °C and mounted with Duolink Mounting Medium after DAPI counterstaining. Positive protein-protein interaction signals were displayed as red fluorescent spots.

### Ethical approval

An approval for this study (prot. n. 620/CE, study n. CE 40/14), carried out in accordance with the declaration of Helsinki as revised in 2013, was granted by the Hospital Research Ethics Committee of Novara (Italy). Board members: President - Roberto Fantozzi; Components - Gian Carlo Avanzi, Maria Angela Brustia, Claudio De Pieri, Roberto Fantozzi, Edoardo Ferlito, Lorenzo Giudice, Gianenrico Guida, Corrado Magnani, Francesco Pia, Alessia Pisterna, Pacifico Uglietti, Libero Zannino.

## Results

### The 3D in vitro epithelial cultures resembled the histology of in vivo tissues

To assess the morphology of the 3D reconstructed epithelia, a H/E staining was performed. The 3D normal and E6E7HPV16 epithelial models completely resemble the typical histology of both in vivo normal and HPV16 infected AINIII epithelia (Fig. 1A-D).

Particularly, in vitro reconstructed and in vivo normal squamous epithelia show the same thickness, organized stratification and keratinization, while in vitro E6E7HPV16 and in vivo HPV16 positive ones are both thicker, with the typical dysplastic multilayered pattern and superficial hyperkeratosis.

### 3D in vitro epithelial cultures

#### HPV16 DNA expression is restricted to BrdU negative cells

To confirm the presence of HPV16 DNA in the 3D epithelial sections, a FISH assay was performed. As shown in Fig. 2B, the HPV16 DNA-FISH specific dot-like punctuate nuclear pattern is clearly visible in the in vitro reconstructed HPV positive 3D model. As in vivo occurs, the staining is distributed inside the epithelium, evidencing the characteristic viral genome amplification, promoted by E6E7 oncoproteins, that occurs mainly in the middle and more superficial layers. No signal is visible in the uninfected in vitro 3D control cultures (Fig. 2A).

The majority of cells showing intense red positive staining for HPV16 DNA is negative for the BrdU signal, indeed strongly present in S phase cells as depicted in Fig. 2D. S phase BrdU positive cells are confined only in the basal layer of the control cultures (Fig. 2C).

#### E6/E7 oncoproteins expression in HPV16 infected 3D epithelia

A direct immunofluorescent analysis for the viral oncoproteins presence was performed. Early E6E7HPV16 oncoproteins are distributed into the nuclei of basal and differentiated epithelial cells as shown in red in Fig. 2F-F’ and H-H′ in the HPV16 infected epithelia, while they are completely absent in normal 3D epithelia (Fig. 2E, G)**.** E6 and E7 oncoproteins expression was confirmed by a western blot analysis (Fig. 2I).

### Characterization of the DDR in the 3D in vitro HPV16 epithelial models

#### γH2A.X and 53BP1 DSBs sensors upregulation in HPV infected 3D epithelia

HPV16 oncoproteins induce DNA damage response as shown by the phosphorylation of the histone variant H2A.X (γH2A.X), which is a sensor of DNA lesions, and by the increase of the number of 53BP1-positive foci with punctuate signals, respect to the normal epithelium (Fig. 3A), in both undifferentiated and differentiated layers (Fig. 3C-C’-C″). Noticeable γH2A.X-positive foci are mainly evident in the mid-upper strata (Fig. 3C). These data were confirmed by a western blot analysis (Fig. 3B).

#### E6 and E7 tissue distribution partially overlaps with that of cyclin E2, B1 and 53BP1

A double immunofluorescent analysis of E6 and E7, together with cyclin E2 and B1 proteins, was made. As noticeable, according to cell cycle analysis, it is possible to observe that E6 and E7 HPV16 increase the proliferation by pushing differentiating cells into G1/S (cyclin E2 positive cells; Fig. 4B for E6/cyclin E2 and 4F for E7/cyclin E2) and S/G2 phases (cyclin B1 positive cells; Fig. 4C for E6/cyclin B1 and 4G for E7/cyclin B1). To gain evidence of the possible interaction of E6 and E7 with 53BP1, we firstly performed immunofluorescent analysis to assess their reciprocal cellular and tissue distribution. CaSki cells show a high number of discrete 53BP1-positive foci inside many E6 and E7 positive cells (Fig. 4A-A’-A” and 4E-E’-E” for E6 and E7 respectively); the same signals are visible in HPV positive models throughout the epithelium (Fig. 4D for E6/53BP1 and 4H for E7/53BP1).

#### The 53BP1 BRCT2 domain contains a leucin rich LXXLL sequence

Sequence analysis [22] evidenced that 53BP1 protein contains a leucin rich LXXLL sequence (LKVLL) located within its BRCT2 domain (^1869^PRENPFQN**LKVLL**VSDQQQN---), hence reinforcing our interaction hypothesis.

#### HPV16 E6 and E7 oncoproteins interact with 53BP1

Upon DNA DSBs, 53BP1 is recruited to the damaged chromosomes to activate the so-called DDR, which culminates with the activation of p53 and pRb. When HPV16 infection occurs, the activation is impaired, since E6 and E7 bind and independently degrade the two cellular tumor suppressors [60]. Based on the evidences above highlighted and on the presence of a LXXLL sequence within 53BP1 BRCT2 domain and of E6 and E7 colocalization with 53BP1 inside CaSki cells and in several cells in the reconstructed epithelium, we hypothesized that E6 and E7 oncoproteins could both interact with 53BP1.

**Fig. 5:**
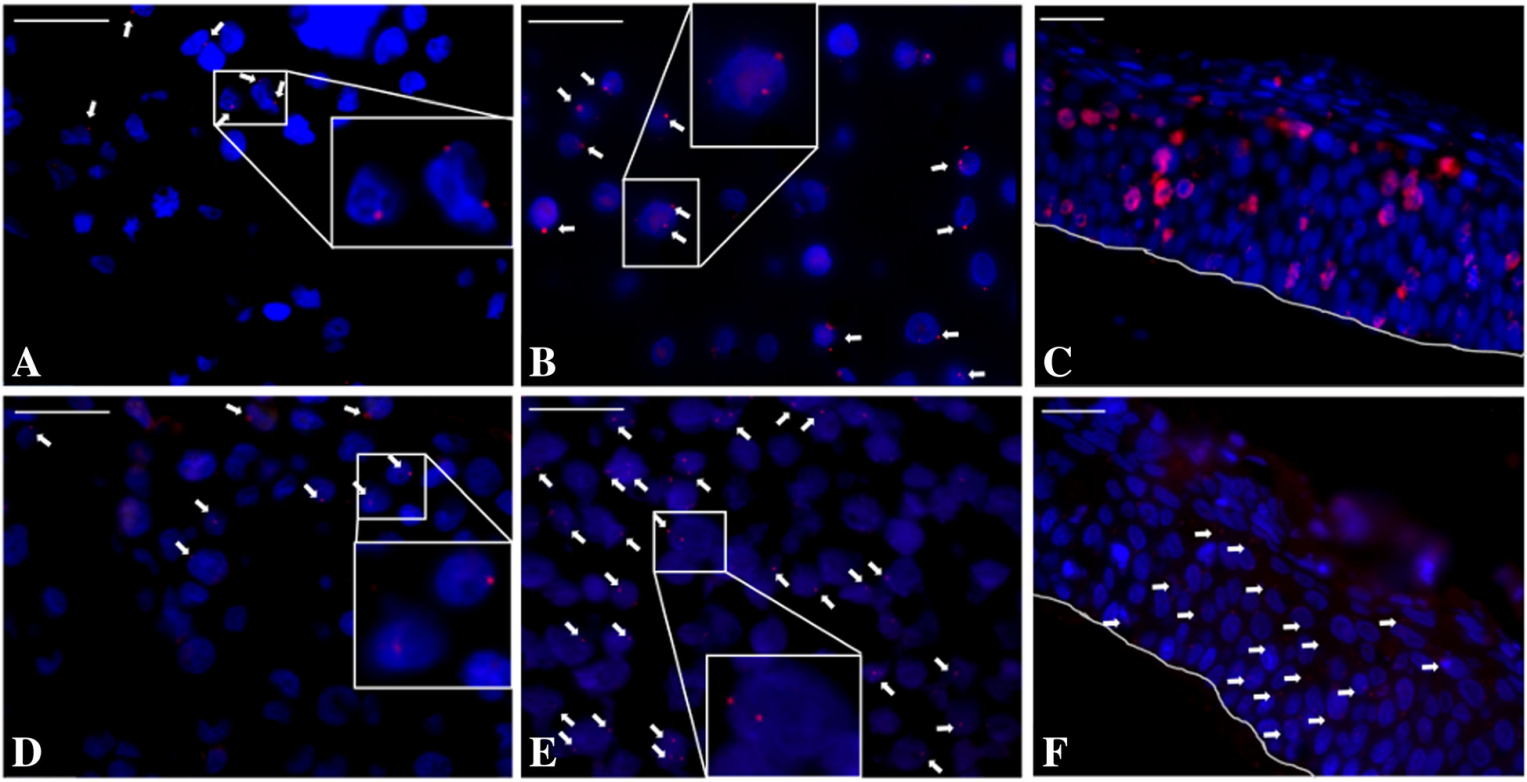
E6 and E7 HPV16 oncoproteins interaction with 53BP1 proteins visualized with PLA technique. The red perinuclear punctuate dots visible in the CaSki (**A**-**D**) and E6E7 HPV16 NHEKs (**B**-**E**) are also present in the HPV16 reconstructed infected epithelium (**C** and **F**). Nuclei were always visualized in blue with DAPI counterstaining. Bar scales in 20x fields = 100 μm

To test this assumption, we investigated, and therefore demonstrated by PLA technique, these bindings both in CaSki and E6E7/HPV16NHEK cells and into the reconstructed infected epithelium. The E6-53BP1 complexes were mainly visible in the perinuclear compartment of both the 2D and 3D models analyzed; the E7-53BP1 ones were also noticeable in the form of smallest punctuate perinuclear signals; moreover in the 3D models, dots were also present in the extracellular milieu, as shown in Figs. 5A, B, C (for E6) and 5D, E, F (for E7). In negative control sections, no signals were observed (data not shown).

## Discussion

Cell replication fidelity is the result of the DDR, a complex signaling proteins network that finds, reports, and repairs lesions that occur to injured DNA. Many DNA viruses actively promote their life cycles through the inactivation of the host DDR, that ordinarily acts by blocking the cell cycle progression [61]. On the contrary, despite the DDR turned on that HR-HPVs have learned to drive, these viruses highly promote the proliferation of the infected squamous epithelial cells. Why and how HR-HPVs activate the DDR through E6 and E7 oncoproteins, thus favoring cancer progression, is still a matter of debate; what it is known is that these events are not so useful for maintaining, but rather for amplifying viral DNA replication.

In particular, during HPV16 infection early phases, E6 oncoprotein manipulates the DDR response through the link to p53 and degradation, mediated by LXXLL E6AP binding motif; unfortunately, a direct role for E6 in the DDR response has not clearly identified yet.

Similarly, E7 increases DDR proteins levels through several main mechanisms: *i)* by competing with E2F1-pRb interaction, thus leading to the inactivation of pRb, and to the promotion, E2F1 mediated, of DDR genes translation, *ii)* by binding to the pRb-like proteins CBP/p300 and p107, that also harbour LXXLL sequence, and *iii)* via the interaction and activation of several DDR proteins, most of them yet unknown.

Therefore, it can be supposed that the DDR is bypassed by the over-expression of the viral oncogenes that promote cell cycle progression. Furthermore, several recent studies sustain the hypothesis that HPV chromatin its-self is modified by the DDR. In particular Gillespie et al. demonstrated that γH2A.X, a marker for cellular response to DNA damage, localizes to HPV replication compartments, inside nuclear foci, whose size directly increases together with virus productive replication [12]. Importantly, γH2A.X was found to link to viral DNA, suggesting the enrollment of cells repair factors into specific viral replication sites. In support of this, DDR components that rely on γH2A.X for recruitment to DNA breaks, including 53BP1, Nbs1, BRCA1, and Rad51, also localize to HPV replication compartments. In particular E7 promotes DNA breaks accumulation inside cells harboring γH2A.X nuclear foci, while, even if E6 can also increase DNA breaks, it seems not to promote γH2AX foci number [14]. To support these evidences, Park et al. [62–64] demonstrated, in genetically engineered murine models expressing E6 and E7 HPV16 oncoproteins, that HPV16 E7, alone or together with E6, was able to promote an accumulation of γH2A.X nuclear foci inside epithelial cells, while E6 alone did not.

In order to add evidences regarding E6 and E7 role in the DDR, we firstly produced an in vitro 3D epithelium, made of HPV16 E6E7 transduced keratinocytes, already consolidated study model for HPVs, and assessed a H&E, BrdU, HPV16 DNA and E6E7 proteins staining.

It is well known that γH2AX is the most suitable DNA damage and repair sensor marker and that 53BP1 protein is one of the DDR components recruited to DNA breaks [65–67]. To ascertain the DDR in the 3D in vitro HPV16 epithelial model, we evaluated both γH2A.X and 53BP1 positive nuclear punctuate signals; they were both present, but not in the normal counterpart, as expected.

Then we evaluated if also cyclins expression was in line with what happens in vivo during HR-HPV infection. As described by the literature, cyclin E is physiologically produced in late G1 until cells enter in S phase and then decreases. Moreover, DNA damaged S cells normally inhibit activation and nuclear import of cyclin B1-Cdk, master regulator of the entry in M phase, therefore stopping in G2 to avoid oncogenic transformation. In DNA damaged HPV16 infected cells these cyclins are no more modulated; indeed they are overexpressed, indicating a clear propensity towards tumoral transformation [68–70]. All these in vivo events, we observed in vitro*,* are displayed in Fig. 4.

Several proteins, such as 53BP1, MDC1, BRCA1, TOPBP1 and Claspin sense the DNA damage. Since 53BP1, like E6 and E7, is a binding partner of the central DNA binding domain of p53, pRb, CBP/p300 and p107 pRb-like proteins, with whom it cooperates in tumor suppression [35–37, 71–73], we hypothesized that E6 and E7 could bind to this DNA damage detector. In support to our hypothesis came the fact that defects in DNA damage control checkpoints are able to promote chromosome translocations and tumorigenesis, particularly in the context in which p53 and pRb dependent apoptosis is abrogated, like in case of E6 and E7 HPV16 overexpression [39].

To gain evidence on our hypothesis, we firstly performed double immunofluorescent and biochemical assays. Comforted by the evidence of the similar punctuate localization pattern of 53BP1 and E6 and E7 proteins in the 3D model (Fig. 4A, A’, A”, D, E, E’, E” and H), we reinforced the association between HPV infection, DDR and genome injury amplification also in CaSki and E6E7HPV16 cells. CaSki, whose HPV16 genome is known to be of about 600 copies of integrated DNA/cell, highly express the E6E7 ORFs [59]. These cells, often used to study chromosomal rearrangements and genomic instability induced by HR-HPV infection and integration, were here utilized as control cells [10, 74].

Martinez-Zapien et al. [23] show that LXXLL motif containing proteins could target E6 and E7; we therefore analyzed 53BP1 sequence and we found a LXXLL (LKVLL) motif within its BRTC2 domain. By comparing this site with those of p53 (LWKLL), we observed a similar total charge. On the contrary, we detected the same total charge among the E6AP- (LQELL), CBP/p300- (LQDLL) and p107 (LDQLL) domains. This analysis let us suppose that hydrophobicity and a slightly positivity of the leucin rich motif could guarantee the optimal conformation to allow the interaction with E6 and E7. Considering that E6 and E7 LXXLL motifs both contain hydrophilic, acid and negative charged aminoacids, we supposed that 53BP1 pocket could enable not only E6- but also E7-53BP1 interactions.

Finally, we performed an in situ PLA technique, which allowed us to detect the direct protein-protein E6-53BP1 and E7-53BP1 interactions within CaSki, E6E7HPV16 NHEKs cells and, more consistently, inside the in vitro reconstructed infected tissue. The PLA signal is different between CaSki (2D) and 3D epithelial cultures; precisely, in CaSki is cytoplasmic/perinuclear, while in 3D is mainly nuclear.

Our data are in agreement with those of Dreier et al.; the subcellular localization of endogenous HPV16 E7 oncoprotein varies during the cell cycle: many interphase (G1, S or G2 phases) CaSki cells showed a predominantly diffuse subcellular cytoplasmic HPV16 E7 expression with a ring structure surrounding the nucleus (that occurs shortly in the early G1 phase), while only few mitotic cells displayed a faint nuclear pattern. It is likely that the nuclear E7 structures reflect well established nuclear E7 functions, such as deregulation of the p16^Ink4A^/pRb pathway.

In the 3D model, E7 is mainly intranuclear where it co-localize with 53BP1 that is present in mitotic cells during telophase and cytokinesis [75].

The E6-53BP1 signal was mainly in the perinuclear compartment in big complexes just where E6 is necessary to degrade p53 [76], while the smallest E7-53BP1 perinuclear punctuate signals are also present in the extracellular milieu of the 3D model, sign of E7 release into the extracellular compartment where it is needed to exert its immunosuppressive role in the in vivo context [77].

These findings were reached thanks to the excellent PLA technology, with its high sensibility and specificity that allows to detect, as distinct spots, and with sub-cellular resolution and molecular precision (0–40 nm), single-molecule protein-protein interaction events. The exponential rolling circle amplification that occurs produces very strong and visible signals, also in case of limited number of interacting molecules. This assay can in situ identify, validate and characterize interactions in native proteins in their correct tissue/cell context under near natural/physiological conditions, without requiring protein content extraction from tissue or the need to over-express the target proteins. Conversely, as also observed by other authors, since co-IP needs high concentrations of both target proteins to generate effective signals and is often dependent on antibodies affinity [42, 43, 52–58], it can’t be the right way to see protein interactions in our 3D models where cellular density and consequently target proteins are not so highly expressed.

In situ PLA has 5-times higher specificity and sensibility than IF [78], that conversely allows the detection of single signals if only at a distance of approximately 0.2 μm.

In conclusion this study highlight, for the first time in our knowledge, the interaction between HPV16 E6 and E7 with the 53BP1 protein that is recruited inside nuclear foci after DNA damage. Further investigations on mutants of the LVKLL motif are necessary to definitively assess if the bindings occur just through this site.

The discovery of such interactions is important not only to better understand proteins function and behavior, specifically of E6 and E7, but also to predict the biological processes and pathways in which those proteins are involved in. In our opinion these interactions could explain why during HPV induced carcinogenesis 53BP1 doesn’t correctly process the DDR signal and doesn’t define DSB repair pathway choice in the G_1_ and S/G_2_ phases.

## Conclusions

In short, we employed a highly sensible and specific PLA assay for E6- and E7-53BP1 interactions detection; our results reinforce once more HPV16 role in cellular function control providing potentially new insights into the activity of this tumor virus.

## Abbreviations

AIN: Anogenital intraepithelial neoplasia
BrdU: 5′-bromo-2′-deoxyuridine
BSA: Bovine serum albumin
co-IP: co-immunoprecipitation
DDR: DNA damage response
DSB: Double-strand break
E6AP: E6-Associated Protein
FBS: Foetal bovine serum
FISH: Fluorescent in situ hybridization
GIN: Genomic instability
H&E: Haematoxylin and eosin
HDFs: Human dermal fibroblasts
HR-HPV: High-risk *Human papillomavirus*
NHEKs: Normal human epidermal keratinocytes
ON: Overnight
PLA: Proximity Ligation Assay
RT: Room temperature
TSA: Tyramide Signal Amplification
WHO: World Health Organization
